# Supplementary material for: Bacterial Microbiota of Ostreobium, the Coral-Isolated Chlorophyte Ectosymbiont, at Contrasted Salinities
Source: Microorganisms. 2023 May 17;11(5):1318. doi: 10.3390/microorganisms11051318 (PMC10223477; doi:10.3390/microorganisms11051318)
Supplement: Supplementary file 1 [file microorganisms-11-01318-s001.zip › microorganisms-2310844-supplementary.pdf]

Supplementary Material

Long-term high salinity exposure protocol for **Ostreobium** *algal strains*

Figure S1: Experimental design for multiple cultures and salinity treatments of two *Ostreobium* strains. m: month(s). Controlled Temperature (25°C). Gradual, monthly acclimatization of each separate culture to increasing salinity in the range 32.9 -40.2 psu.

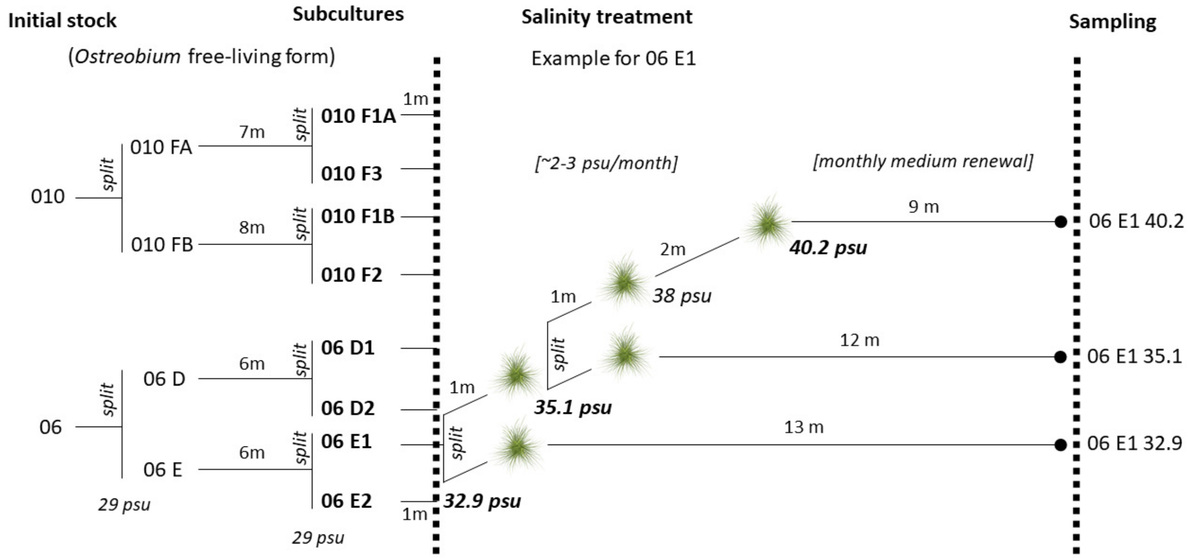

Comparison of bacterial associates from cultured **Ostreobium** *to field occurring Ostreobium-bacteria assemblages*

To contextualize the culture-based algal microbiota diversity relative to the complex, naturally occurring, endolithic diversity in coral skeletons, we analyzed the diversity of *Ostreobineae* and bacterial communities in environmental *Pocillopora* sp. coral skeletons (as the studied *Ostreobium* strains were initially isolated from cultured *Pocillopora acuta* corals). The endolithic bacterial diversity of wild populations of corals is sensitive to fluctuating environmental conditions, including salinity but also temperature and turbidity, in contrast to the controlled stable conditions of laboratory-based experiments, where only salinity was manipulated. However, comparison between environmental and cultured samples allowed assessment of the bacterial fraction persistently associated to *Ostreobium*, i.e. conserved since isolation and across multiple algal subcultures, and hence of potential biological importance for the algal host.

DNA extraction of environmental coral skeletons

Skeleton fragments of environmental *Pocillopora* sp. corals collected at a medium-low salinity reef site, 34.4 psu and 28.5°C (Guam, Pacific, n=4), and a high salinity reef site, ~40.6 psu and 26°C (Eilat, Red Sea, n=4), were used as controls for wild populations of *Ostreobium* and associated bacteria, with microboring Ostreobineae presence confirmed by *tuf*A and *rbc*L amplicon sequencing (see below). Skeletal DNA was extracted after removal of coral tissues (Waterpick® method), using Quiagen DNeasy PowerSoil™ Kit after protocol from Massé *et al.* [14]. For each reef site, multiple colonies (n=4) were sampled. Skeletons of *Pocillopora* sp. *cf meandrina* corals from Guam island site 2 (13.42 N 144.64 E) are part of the Tara Pacific 2016-2018 collection (M152C01/CO-1008013, M152C02/CO-1008014, M152C05/CO-1008017, M152C06/CO-1008018) with salinity and temperature indicated in Table S2. Skeletons of *Pocillopora* sp. *cf verrucosa* colonies from Eilat InterUniversity Institute (IUI) reef nursery

table (34.92 N 29.50 E) (P1, P2, P3, P4) were collected at depth 6-10m on July 4 2019 during the European Union's Horizon2020 ASSEMBLE Plus (grant N°730984) 2018 Transnational Access project CORALBOUCLE-CLIM (collection permit 209/42300 Israel Nature and Parks Authority to M. Fine), with salinity and temperature retrieved from the Israel National Monitoring Program at the Gulf of Eilat datasets.

PCR amplification, Illumina sequencing and bioinformatics workflow for environmental coral skeletons

The bacterial 16S rDNA V5-V7 region (~400 bp) was amplified from skeletal DNA extracts, sequenced, and analyzed according to the protocol described for *Ostreobium* bacterial communities (see main text and Table S1).

Table S1: (A) Read counts of 16S rDNA ASVs obtained from cultured *Ostreobium* thalli, their supernatants, coral skeletons and internal controls using Illumina MiSeq sequencing (unfiltered data). (B) Read counts of bacterial 16S rDNA ASVs after removing ASVs affiliated to eukaryota, chloroplast, mitochondria, unassigned ASVs and all ASVs of the internal controls (filtered data). (C) Fasta sequences of 16S rDNA ASVs (unfiltered data). (D) Taxonomy of bacterial 16S rDNA ASVs (filtered data). (E) Fasta sequences of Ostreobineae *tuf*A ASVs detected in coral skeletons from Guam (Pacific) and Eilat (Red Sea). This Supplementary Table 1 is available on figshare with doi: 10.6084/m9.figshare.21953075.

Ostreobium diversity was detected in skeletal DNA extracts by amplicon-sequencing of the *rbc*L gene fragment with the primers *rbc*LF250 ([5' GATATTGARCCTGTTGTTG GTGAAGA 3'] ; modified from Gutner-Hoch and Fine [81]) and *rbc*L1391R [82] (as previously published in Massé *et al.* [14]), or the *tuf*A elongation factor gene fragment with primer pair: Oq-tuf [80] / *tuf*AR [83] (~490 bp) and *tubryo*F / *tubryo*R (~600 bp, [5]). Additionally, the *tuf*A marker was amplified from DNA extracts of thalli of *Ostreobium* strain 06 and 010 with both primer pairs. Amplicons were submitted for MiSeq sequencing (Illumina, paired-end) to the MNHN SSM (Service de Systématique Moléculaire) platform (MNHN-CNRS UAR2700 'Acquisition et Analyse de Données pour l'Histoire naturelle').

Paired-end reads of *tuf*A were assembled and analyzed using an in-house bioinformatics pipeline. Briefly, primers were removed from the raw reads using CUTADAPT and DADA2 was used for quality filtering and identification of ASVs [84]. A table of ASVs across different samples was generated. Plastid sequences were then aligned by local BLASTn with *tuf*A-encoding sequences available in GenBank database (consulted on 2 May 2022) for classification to *Ostreobium* families *sensu* Sauvage *et al.* [5].

Ostreobium *diversity in environmental* Pocillopora *sp. coral skeletons*

Pocillopora coral skeletons from Guam (Pacific; 34.4 psu, 28.5°C) and Eilat (Red Sea; ~40.6 psu, 26°C) contained Ostreobineae, confirmed via *rbc*L and *tuf*A sequence analysis, summarized in Table S2. In *Pocillopora* from Guam, *Ostreobium* was detected in all four colony replicates and all ASVs were affiliated to the Odoaceae family based on *tuf*A marker sequences (*sensu* Sauvage *et al.* [5]; >85% sequence similarity to the designated "type" strain). In *Pocillopora* from Eilat, *Ostreobium* ASVs were detected in 3 out of 4 colonies using the *tuf*A marker. In one coral colony two *Ostreobium* families co-occurred, the Maedaceae and the Odoaceae (*sensu* Sauvage *et al.* [5]), as well as ASVs unclassified at family level (nucleotide sequence divergence >15%). In two other coral colonies, ASVs affiliated either to the Odoaceae family or unclassified at family level were detected.

In *Ostreobium* strains, *tuf*A sequences were amplified using both molecular markers but only the *tubryo*F/*tubryo*R primers allowed to retrieve *tuf*A sequences of Ostreobineae. For strain 06, its Ostreobineae ASV was affiliated to the family Hamidaceae; *sensu* Sauvage *et al.* [5]. For strain 010, all retrieved *tuf*A sequences classified to non-targeted bacterial taxa; however, although *tuf*A family-level classification of this strain was not possible, this strain was affiliated to the Ostreobineae suborder by previous *rbc*L sequencing [7].

Table S2: *Ostreobium* phylotypes detected in coral skeletons of living colonies of *Pocillopora* sp. from Guam (Pacific) and Eilat (Red Sea). Metabarcoded sequences of two plastidic markers were obtained: *rbc*L with primers *rbc*L250F [7]/*rbc*L1391R [82] and *tuf*A with either the primers Oq-tuf [80]/tufAR [83] or *tu*bryoF/*tu*bryoR [5]. Affiliation to '*Ostreobium*' families (*sensu* Sauvage *et al.* [5]) was based on *tuf*A sequences with sequence similarity >85% to the designated "type" strain for each family (unclassified if similarity <85%) (family-level affiliation of *rbc*L sequences are not delimited.) Sequences of *tuf*A ASVs are provided in Table S1. Fasta sequences of Ostreobineae *tuf*A ASVs detected in coral skeletons (Table S2) were deposited at figshare with doi: 10.6084/m9.figshare.21953075 (Table S1).

Site	Salinity (psu)	T (°C)	*Pocillopora sp.* colony	Marker	Primer pairs	Ostreobineae ASVs	*Ostreobium* family (AVSs)	ASV ID
Guam (Pacific)	34.4	28.5	M152CO1	*rbc*L	*rbc*L250F/*rbc*L1391R	10	NA	NA
				*tuf*A	Oq-tuf/*tufA*R	3	Odoceae (3)	ASV_215; ASV_226; ASV_419
				*tuf*A	*tu*bryoF/*tu*bryoR	16	Odoceae (16)	ASV_24; ASV_28; ASV_32; ASV_55; ASV_90; ASV_116; ASV_163; ASV_218; ASV_232; ASV_266; ASV_272; ASV_308; ASV_335; ASV_349; ASV_360; ASV_438
Guam (Pacific)	34.4	28.5	M152CO2	*rbc*L	*rbc*L250F/*rbc*L1391R	9	NA	NA
				*tuf*A	Oq-tuf/*tufA*R	0	/	/
				*tuf*A	*tu*bryoF/*tu*bryoR	19	Odoceae (19)	ASV_16; ASV_25; ASV_30; ASV_32; ASV_54 ; ASV_75; ASV_88; ASV_106; ASV_117; ASV_134 ; ASV_171; ASV_176; ASV_267; ASV_322; ASV_350; ASV_381; ASV_398; ASV_414; ASV_429
Guam (Pacific)	34.4	28.5	M152CO5	*rbc*L	*rbc*L250F/*rbc*L1391R	2	NA	NA
				*tuf*A	Oq-tuf/*tufA*R	0	/	/
				*tuf*A	*tu*bryoF/*tu*bryoR	10	Odoceae (10)	ASV_16; ASV_138; ASV_141; ASV_171; ASV_195; ASV_268; ASV_273; ASV_276; ASV_315; ASV_415
Guam (Pacific)	34.4	28.5	M152CO6	*rbc*L	*rbc*L250F/*rbc*L1391R	3	NA	NA
				*tuf*A	Oq-tuf/*tufA*R	1	Odoceae (1)	ASV_1115
				*tuf*A	*tu*bryoF/*tu*bryoR	2	Odoceae (2)	ASV_32; ASV_457
Eilat (Red Sea)	~40.6	26	P1	*rbc*L	*rbc*L250F/*rbc*L1391R	NA	NA	NA
				*tuf*A	Oq-tuf/*tufA*R	1	unclassified Ostreobiaceae (1)	ASV_2126
				*tuf*A	*tu*bryoF/*tu*bryoR	0	/	/
Eilat (Red Sea)	~40.6	26	P2	*rbc*L	*rbc*L250F/*rbc*L1391R	NA	NA	NA
				*tuf*A	Oq-tuf/*tufA*R	0	/	/
				*tuf*A	*tu*bryoF/*tu*bryoR	29	Maedaceae (11) Odoceae (14) unclassified Ostreobiaceae (4)	ASV_1 ; ASV_9 ; ASV_39; ASV_48b; ASV_51; ASV_57 ; ASV_63; ASV_83; ASV_86 ; ASV_87; ASV_98; ASV_102; ASV_126; ASV_162; ASV_174; ASV_175; ASV_184; ASV_187; ASV_239; ASV_251b; ASV_314; ASV_321; ASV_357; ASV_396 ; ASV_405; ASV_406; ASV_407; ASV_413; ASV_436
Eilat (Red Sea)	~40.6	26	P3	*rbc*L	*rbc*L250F/*rbc*L1391R	NA	NA	NA
				*tuf*A	Oq-tuf/*tufA*R	0	/	/
				*tuf*A	*tu*bryoF/*tu*bryoR	0	/	/
Eilat (Red Sea)	~40.6	26	P4	*rbc*L	*rbc*L250F/*rbc*L1391R	NA	NA	NA
				*tuf*A	Oq-tuf/*tufA*R	4	Odoceae (4)	ASV_2051; ASV_2122; ASV_2168; ASV_2330
				*tuf*A	*tu*bryoF/*tu*bryoR	17	Odoceae (17)	ASV_20; ASV_22; ASV_23; ASV_27; ASV_31; ASV_35; ASV_47; ASV_48a; ASV_58; ASV_64; ASV_115; ASV_149; ASV_156; ASV_160; ASV236; ASV_251a; ASV_658

Diversity and structure of bacterial communities in Ostreobium-*colonized* Pocillopora *skeletons, compared to cultured* Ostreobium

In environmental corals, i.e., complex endolithic assemblages of *Pocillopora* sp. from Guam (Pacific) and Eilat (Red Sea) including diverse Ostreobineae phylotypes (see above and Table S2), a total of 1,147,905 Illumina MiSeq sequences of the V5-V7 region of bacterial 16S rDNA were obtained. After removal of ASVs affiliated to Chloroplast (4 ASVs), Mitochondria (7 ASVs) and Unassigned sequences (7 ASVs) and of the internal controls (all 1,617 ASVs detected in 9 controls, no matter their relative abundances, including 144 ASVs shared between controls and at least one coral skeleton sample), a cumulated total of 2,412 cumulated bacterial ASVs were detected (total n=8 skeletons) averaging 259±61 ASVs per sample for Guam and 423±141 for Eilat. Rarefaction curves of environmental coral skeletons did not flatten out (Figure S2), in contrast to those of cultured samples, suggesting that additional rare bacterial taxa are probably present but undetected in these *Pocillopora* skeletons.

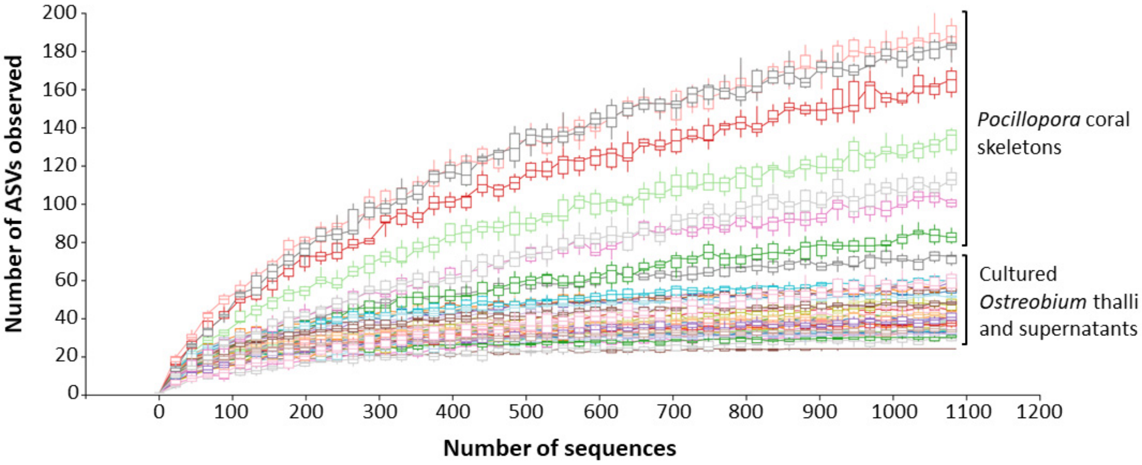

Figure S2: Rarefaction curves from V5-V7 region of bacterial 16S rDNA amplified from cultured *Ostreobium* thalli and supernatants, and environmental *Pocillopora* coral skeletons. Curves were constructed using the rarefied dataset, after filtering out contamination (internal) controls.

The bacterial ASVs richness detected within "wild" *Ostreobium*-colonized *Pocillopora* skeletons from Guam and Eilat was 5-8 folds higher than within cultured *Ostreobium* thalli (both strains 010 and 06; Table S3). A total of 45 bacterial ASVs were shared between *Ostreobium* thalli and environmental corals (5.4% of thalli's ASVs, 1.8% of coral ASVs; Figure S3A), indicating partial overlap of bacterial diversity between the microbiota of cultured and "wild" *Ostreobium* (also visualized by PCoA analyses of community structures, see below Figure S3B). Within coral skeletons, the Pielou's evenness index of bacterial communities was low and highly variable across biological replicates, with average values of 0.48±0.19 in *Pocillopora* sampled at Guam (Pacific) and 0.61±0.10 in *Pocillopora* from Eilat (Red Sea) (Table S3).

Table S3: Raw data of bacterial ASV's richness, Shannon and Pielou's evenness in cultured *Ostreobium* thalli and supernatants, and environmental *Pocillopora* coral skeletons (V5-V7 region of 16S rDNA). Alpha diversity indices were calculated using the unrarefied ASV dataset. Site: geographical origin of sample (ATPD: aquarium-grown colonies of *Pocillopora acuta*); Colony: coral host individual from which *Ostreobium* strains 010 and 06 were isolated or natural endolithic assemblages were characterized.

Sample	Strain	Subculture	*Pocillopora* colony	Site	Salinity and Temperature	16S rDNA sequences	ASV richness (S)	Shannon Index (H)	Pielou's evenness Index (J)
***Ostreobium* algal thalli**	010	F1A	*Pocillopora acuta* Pd2	ATPD	32.9 psu 25°C	4013	59	3.12	0.76
		F1B				4266	64	3.24	0.78
		F2				2514	48	3.00	0.77
		F3				2625	49	3.24	0.83
		F1A			35.1 psu 25°C	2545	39	2.81	0.77
		F1B				4053	45	2.25	0.59
		F2				2283	32	2.80	0.81
		F3				1080	24	2.52	0.79
		F1A			40.2 psu 25°C	5143	64	3.24	0.78
		F1B				3815	42	2.95	0.79
		F2				11703	78	2.82	0.65
		F3				3150	41	2.99	0.81
***Ostreobium* algal thalli**	06	D1	*Pocillopora acuta* Pd2	ATPD	32.9 psu 25°C	17251	51	1.28	0.32
		D2				14564	43	1.40	0.37
		E1				4183	37	2.28	0.63
		E2				2874	35	1.98	0.56
		D1			35.1 psu 25°C	8004	57	2.28	0.56
		D2				14529	53	1.87	0.47
		E1				11101	45	2.21	0.58
		E2				23683	74	1.85	0.43
		D1			40.2 psu 25°C	7183	91	3.00	0.67
		D2				6805	57	2.49	0.62
		E1				9149	51	2.65	0.67
		E2				18723	58	1.79	0.44
***Ostreobium* culture supernatants**	010	F1A	/	/	32.9 psu 25°C	3535	42	2.65	0.71
		F1B	/	/		1315	39	3.00	0.82
		F2	/	/		2097	39	2.88	0.78
		F3	/	/		2298	44	2.51	0.66
		F1A	/	/	35.1 psu 25°C	4601	45	2.61	0.69
		F1B	/	/		5208	42	2.41	0.64
		F2	/	/		2033	39	2.80	0.77
		F3	/	/		3332	68	3.08	0.73
		F1A	/	/	40.2 psu 25°C	5906	68	2.82	0.67
		F1B	/	/		3767	48	2.89	0.75
		F2	/	/		24614	93	1.73	0.38
		F3	/	/		2380	54	3.24	0.81
		D1	/	/		2473	34	2.50	0.71
		D2	/	/		2708	45	2.99	0.79

***Ostreobium* culture supernatants**	06	E1	/	/	32.9 psu 25°C	3732	42	2.45	0.66
		E2	/	/		1673	32	2.50	0.72
		D1	/	/	35.1 psu 25°C	2375	42	2.70	0.72
		D2	/	/		2568	31	2.65	0.77
		E1	/	/		5958	47	2.42	0.63
		E2	/	/		2899	51	2.88	0.73
		D1	/	/	40.2 psu 25°C	2357	38	2.67	0.73
		D2	/	/		1487	34	2.90	0.82
		E1	/	/		6741	50	1.96	0.50
		E2	/	/		2509	36	2.71	0.76
**Environmental *Pocillopora* coral skeletons**	/	/	*Pocillopora* sp. #1	Guam	34.4 psu 28.5°C	51011	174	2.48	0.48
	/	/	*Pocillopora* sp. #2			83904	318	1.32	0.23
	/	/	*Pocillopora* sp. #5			44746	263	3.88	0.70
	/	/	*Pocillopora* sp. #6			148297	281	2.94	0.52
	/	/	*Pocillopora* sp. #1	Eilat	~40.6 psu 26°C	109036	237	2.62	0.48
	/	/	*Pocillopora* sp. #2			86087	405	3.50	0.58
	/	/	*Pocillopora* sp. #3			90618	481	4.07	0.66
	/	/	*Pocillopora* sp. #4			70061	569	4.48	0.71

Figure S3. (A) Venn diagram of bacterial ASV distribution across cultured *Ostreobium* thalli, supernatants, and environmental *Pocillopora* coral skeletons. (B) Structure of bacterial communities visualized by Principal Coordinate analysis of Bray-Curtis distances (calculated using arcsine square root transformed proportions from unrarefied dataset). A great dispersion of microbiota profiles is visible. Differentiation of the microbiota of field coral samples relative to the culture-based samples (thalli and corresponding supernatants) is visible on Component 2. However, partial overlap is detected on Component 1 between the beta-diversity of communities of cultured *Ostreobium* thalli and their supernatants with those of coral skeletons. Differences between cultured and environmental categories were statistically significant (pairwise "adonis" comparisons after permutational multivariate analysis of variance, F=3.649, df1, p=0.001; Table S4A).

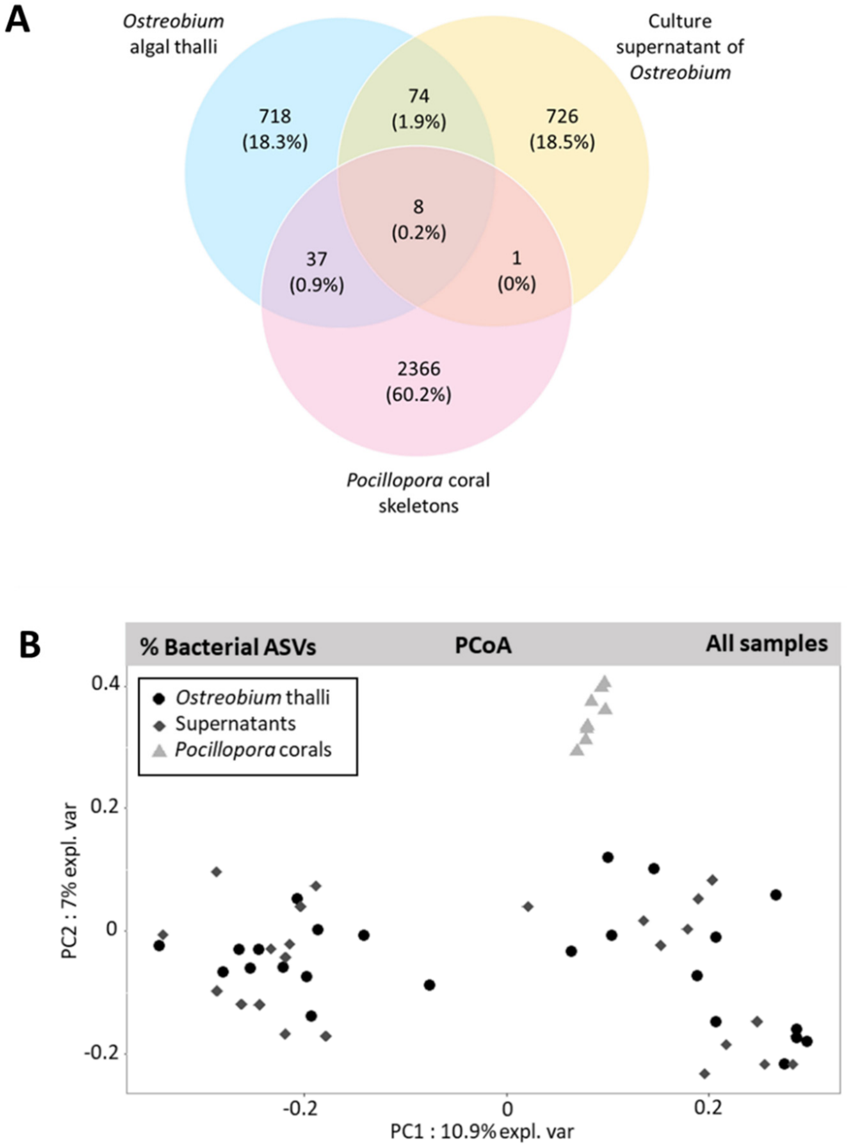

Table S4: Summary of pairwise permutational multivariate analysis of variance ('adonis' function) of Bray-Curtis distances (n=999 permutations, $\alpha = 0.05$) for pairs of factor levels. Bray-Curtis distances were calculated from arcsine square root transformed unrarefied bacterial ASV proportions. (A) Variance comparison between environmental *Pocillopora* coral skeletons *vs. in vitro* algal cultures (*Ostreobium* thalli and supernatants). (B) Variance comparison between cultured algal categories (*Ostreobium* thalli *vs.* supernatants), genetic lineages (010 *rbc*L clade P1 *vs.* 06 *rbc*L clade P14), salinities (32.9 *vs.* 35.1 *vs.* 40.2 psu) and their interactions. (C) Pairwise comparisons of variance within algal cultures for the salinity factor (p-adjusted with Bonferroni correction).

A) Variance comparison between environmental coral skeletons and *in vitro* algal cultures.

	Df	SumOfSqs	R2	F	Pr(>F)
**metadata$treatment**	1	1.4467	0.0633	3.6490	0.001*
**Residual**	54	21.4092	0.9367	NA	NA
**Total**	55	22.8559	1	NA	NA

B) Variance comparison between cultured algal sample categories/nature (thalli *vs.* supernatants), genotype (010: *rbc*L clade P1 *vs.* 06: *rbc*L clade P14), salinity (32.9, 35.1, 40.2 psu) and their interactions.

	Df	SumOfSqs	R2	F	Pr(>F)
**metadata$nature**	1	0.3317	0.0182	0.9730	0.467
**metadata$genotype**	1	1.0393	0.0571	3.0488	0.001*
**metadata$salinity**	1	1.8850	0.1036	5.5297	0.001*
**metadata$salinity:metadata$nature**	1	0.2535	0.0139	0.7438	0.928
**metadata$salinity:metadata$genotype**	1	0.6428	0.0353	1.8855	0.002*
**metadata$genotype:metadata$nature**	1	0.2089	0.0115	0.6127	0.994
**metadata$salinity:metadata$genotype:metadata$nature**	1	0.2069	0.0113	0.6069	0.998
**Residual**	40	13.6357	0.7491	NA	NA
**Total**	47	18.2037	1	NA	NA

C) Pairwise comparison of salinity factor in *in vitro* algal cultures.

	pairs	Df	SumsOfSqs	F.Model	R2	p.value	p.adjusted
1	32.9 *vs* 35.1	1	0.8827	2.5467	0.0782	0.001*	0.003
2	32.9 *vs* 40.2	1	1.9276	5.8334	0.1628	0.001*	0.003
3	35.1 *vs* 40.2	1	1.0054	2.7398	0.0837	0.001*	0.003

***Taxonomic composition of bacterial communities in* Ostreobium*-colonized* Pocillopora *skeletons, compared to cultured* Ostreobium.**

Alphaproteobacteria were detected both in cultures of algal strains and in all *Pocillopora* coral skeletons (Figure S4). The Kiloniellales order, abundant in *Ostreobium* thalli of strain 010 (clade P1) (33.3±16%, n=12) was also detected in skeletons but at 6-fold lower relative abundances of 5.1±6.4% (n=8) (Figure 5 and Figure S5). The Rhodospirillales order, abundant in *Ostreobium* thalli of strain 06 (clade P14) (36.8±19.9%, n=12) was also detected in *Pocillopora* coral skeletons but at trace levels (1.0±2.4%, detected in 6/8 samples). The Bacteroidia class, more abundant in culture supernatants than thalli, was also detected in all coral skeletons, at highly variable abundance (12.1±19.8%, n=8). Within the Gamma-proteobacteria, both Alteromonadales and Cellvibrionales orders were also detected, although at lower abundances than in cultured thalli, in all coral skeletons (n=8, 0.3±0.3% for Alteromonadales and 2.4±5.4% for Cellvibrionales). Acidimicrobiia (represented by the Microtrichales order) and Phycisphaerae (represented by the Phycisphaerales order) were also detected within coral skeletons. The Thermoleophilia class (represented by the Gaiellales order), persistent in thalli of strain 010, was detected only at trace levels in a few coral skeletons (3/8, <0.5%). In contrast to algal cultures, higher diversity, and abundances of Deltaproteobacteria were detected in coral skeletons, with representatives of four orders (Polyangiales, Myxococcales, Leptospirales and Desulfovibrionales) and higher cumulated relative abundances (6.8±12.7%, n=8), instead of only one order (Nannocystales) in thalli of *Ostreobium* strain 010.

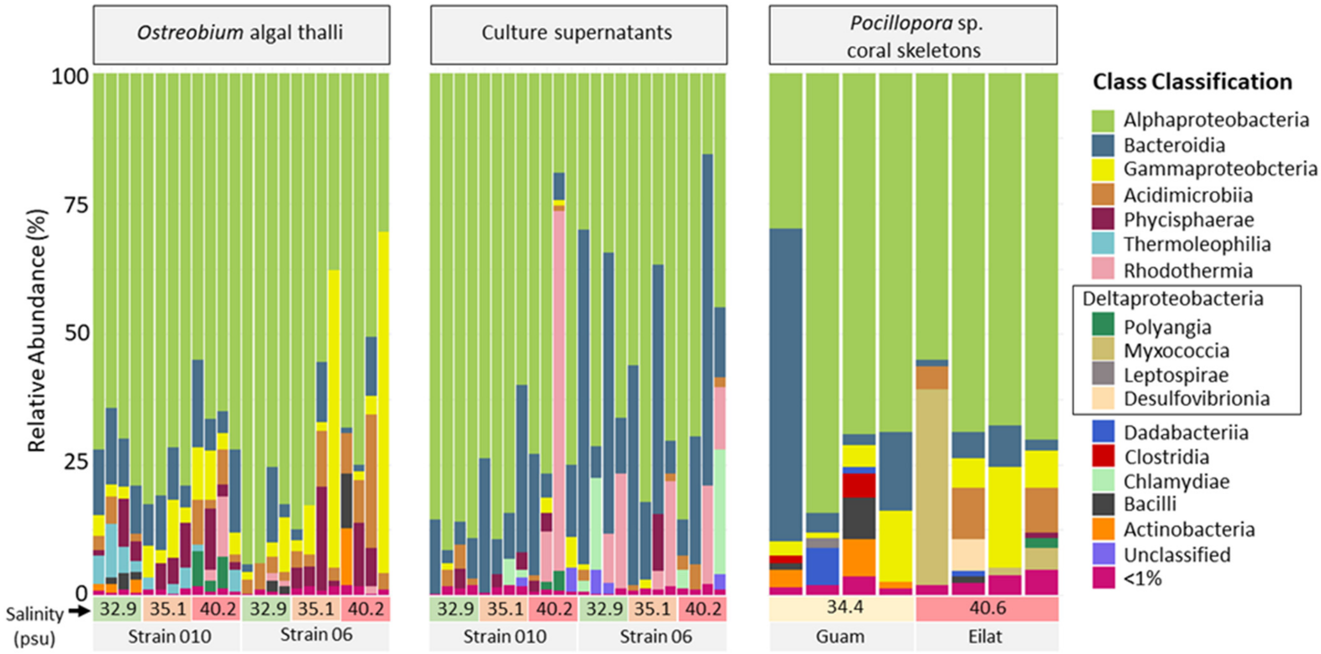

Figure S4: Taxonomic composition of bacterial classes in cultured *Ostreobium* thalli and supernatants, and the environmental *Pocillopora* coral skeletons. Salinity and algal genotype or reef geography are indicated below each individual sample column. Abiotic factors other than salinity fluctuate between coral field samples, affecting microbiota composition. Classes detected at trace levels were aggregated in the <1% abundance category.

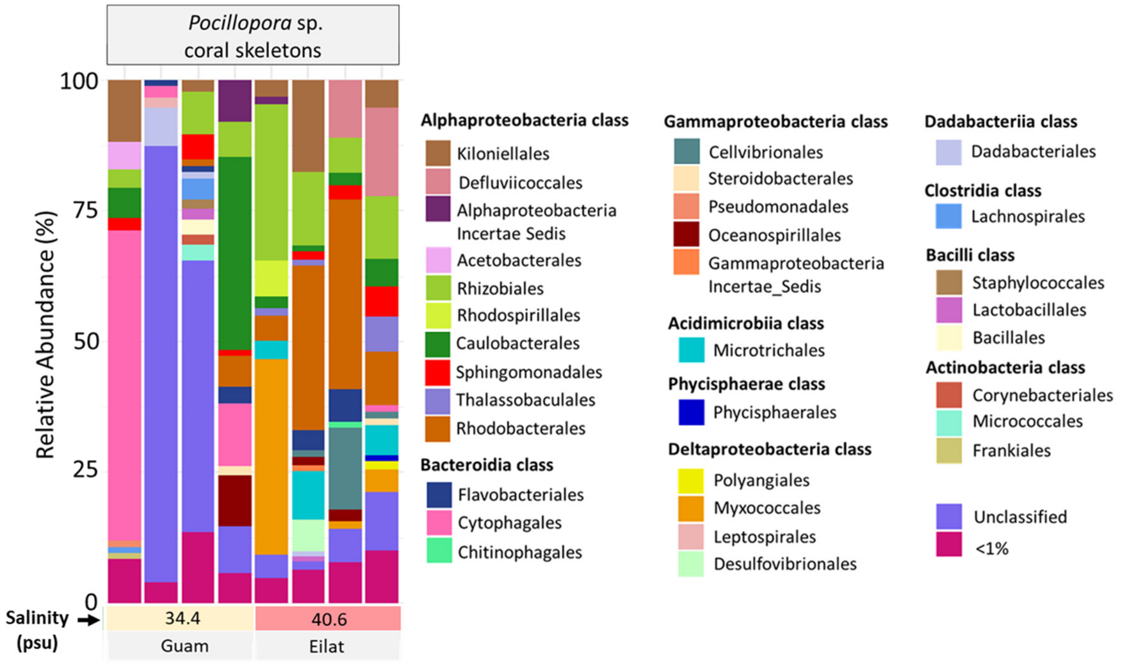

Figure S5. Taxonomic composition of bacterial orders *Pocillopora* coral skeletons. Salinity and reef geography are indicated below each individual sample column. Abiotic factors other than salinity fluctuate between coral field samples, affecting microbiota composition. Among the 134 bacterial orders, those detected at trace levels were aggregated in the <1% abundance category.

Detection of core bacteria from cultured **Ostreobium** *within bacterial communities in* **Ostreobium**-*colonized* **Pocillopora** *skeletons.*

Our results highlight partial overlap between environmental *Pocillopora* coral endolithic bacteria and bacteria co-cultured with *Ostreobium* strains, initially isolated from *Pocillopora acuta*. The classification of shared ASVs, and their average relative abundances and prevalence among algal thalli, supernatants, and environmental coral skeletons, are detailed in Table S5.

In coral-associated endolithic bacterial assemblages, systemic surveys highlight the co-occurrence of prokaryotes with several *Ostreobium* genotypes together with fungal and protists microeukaryotes [23, 25, 85]. Here, we present new data on skeleton bacteria from Red Sea (Eilat) and central Pacific (Guam) *Pocillopora* sp. corals, completing previous surveys of *Pocillopora damicornis* from Papua New Guinea [26] and from the Great Barrier Reef [54]. Our results confirm the skeletal presence although at low abundances of the genus *Endozoicomonas* (Gammaproteobacteria; 14 ASVs detected, 1.2% cumulated abundances), which is a prevalent symbiont in many corals [86]. *Endozoicomonas* is especially abundant and persistent within tissues of Red Sea *Pocillopora verrucosa* (2 phylotypes, 90% cumulated abundance; [87]). It was detected but at much lower abundances in the skeleton of *Pocillopora damicornis* [26, 54] and 9 other scleractinian species (where it represented 7% of skeleton ASVs *vs.* 33% of tissue ASVs across the whole data set; [54]). Here, its absence of detection in a total of 24 cultures from 2 *Ostreobium* genotypes, in parallel to its reported absence from cultured Symbiodiniaceae strains [48, 49, 73] suggest that *Endozoicomonas* may be associated to the coral host and not its microalgal symbionts.

Table S5 : Detailed core *Ostreobium* microbiota composition. Relative abundances (mean ± SD) and prevalence (total detected / total screened samples) of shared bacterial ASVs in both *Ostreobium* genotypes (06 and 010), compared to corresponding supernatants and environmental *Pocillopora* coral skeletons. Taxonomy classification after SILVA v138 SSU rRNA database released December 16, 2019 (bacterial sequences unassigned below the class level were also aligned by BLASTn with 16S RNA-encoding sequences available in GenBank database). NA : unassigned taxonomy.

	Core ASVs	Phylum	Class	Order	Family	Genus	% 06 microbiota (prevalence)	% 010 microbiota (prevalence)	% *Pocillopora* endolithic assemblage (prevalence)	% Culture supernatants 06 (prevalence)	% Culture supernatants 010 (prevalence)
**Core microbiota** (ASVs shared across 3 salinities and both genotypes)	**ASV16**	**Proteobacteria**	**Alphaproteobacteria**	**Rhodospirillales**	**Rhodospirillaceae**	**nd**	**32.2± 19.4 (12/12)**	**3.09± 3.08 (9/12)**	< 0.01 (1/8)	2.82±2.08 (12/12)	0.17±0.28 (4/12)
	**ASV124**			**Kiloniellales**	**Fodinicurvataceae**	***Fodinicurvata***	**0.24± 0.39 (4/12)**	**1.87± 1.61 (8/12)**	absent (0/8)	3.5±1.69 (11/12)	1.29±2.16 (4/12)
	**ASV57**			**Caulobacterales**	**Hyphomonadaceae**	**nd**	**1.75±1.83 (10/12)**	**5.67±6.59 (9/12)**	0.01± 0.03 (1/8)	0.75±1.22 (7/12)	1.73±2.46 (8/12)
	**ASV72**			**Rhizobiales**	**Stappiaceae**	***Labrenzia***	**0.65±0.65 (11/12)**	**3.22±6.24 (11/12)**	absent (0/8)	0.19±0.40 (4/12)	1.73±3.16 (8/12)
	**ASV76**			**Rickettsiales**	**AB1**	**AB1**	**0.27±0.33 (6/12)**	**1.66±1.32 (12/12)**	0.01±0.02 (1/8)	0.67±0.81 (6/12)	9.87±7.92 (12/12)
	**ASV166**	**Bacteroidota**	**Bacteroidia**	**Cytophagales**	**Amoebophilaceae**	***Candidatus Amoebophilus***	**0.29±0.23 (12/12)**	**1.18±0.90 (12/12)**	**0.18±0.23 (6/8)**	**<0.01 (1/12)**	**0.02±0.06 (1/12)**
	**ASV167**	**Actinobacteriota**	**Acidimicrobiia**	**Microtrichales**	**Ilumatobacteraceae**	***Ilumatobacter***	**0.27± 0.48 (6/12)**	**1.86±2.17 (9/12)**	absent (0/8)	0.01±0.05 (1/12)	0.61±0.84 (7/12)

**Strain-specific core microbiota** (ASVs shared	**ASV273**	Proteobacteria	Alphaproteobacteria	**Rhodospirillales**	**Rhodospirillaceae**	**nd**	***absent***	***1.77±2.65 (4/12)***	**absent (0/8)**	absent (0/12)	absent (0/12)
	**ASV162**					**nd**	***1.78±3.64 (3/12)***	**** 0.33±1.13 (1/12)***	**absent (0/8)**	0.13±0.46 (1/12)	0.08±0.29 (1/12)
	**ASV244**					**nd**	***0.60± 0.98 (4/12)***	***absent (0/12)***	**absent (0/8)**	absent (0/12)	absent (0/12)
	**ASV150**			**Kiloniellales**	**Fodinicurvataceae**	***Fodinicurvata***	*** 0.12±0.3 (2/12)**	***2.38±2.82 (6/12)***	**absent (0/8)**	0.66±1.76 (2/12)	1.07±3.71 (1/12)
	ASV85				**Kiloniellaceae**	***Pelagibius***	*** 0.59±0.90 (4/12)**	**3.69±3.34 (8/12)**	**absent (0/8)**	1.20±2.44 (3/12)	4.71±2.48 (11/12)
	ASV52					***Pelagibius***	**absent (0/12)**	**9.95±14.90 (6/12)**	**absent (0/8)**	absent (0/12)	8.26±12.9 (4/12)
	ASV101					***Pelagibius***	**0.39±0.68 (4/12)**	**absent (0/12)**	**0.60±1.70 (1/8)**	absent (0/12)	absent (0/12)
	ASV176					**nd**	**absent (0/12)**	**0.98 +/- 0.90 (10/12)**	**absent (0/8)**	absent (0/12)	2.98±2.43 (12/12)
	ASV700			**nd**	**nd**	**nd**	**absent (0/12)**	**0.61±0.68 (8/12)**	**absent (0/8)**	absent (0/12)	0.41±0.57 (8/12)
	ASV110			Rhizobiales	Ahrensiaceae	nd	absent (0/12)	5.06±5.99 (9/12)	absent (0/8)	absent (0/12)	1.02±1.02 (7/12)
	ASV121				Devosiaceae	*Devosia*	1.16±2.16 (7/12)	absent (0/12)	absent (0/8)	1.26 +/- 3.72 (2/12)	absent (0/12)
	ASV35			Rhodobacterales	Rhodobacteraceae	nd	11.67±21.42 (6/12)	absent (0/12)	absent (0/8)	1.03±1.75 (5/12)	absent (0/12)
	ASV1480					*Paracoccus*	0.07±0.13 (5/12)	* 0.03 +/- 0.11 (2/12)	< 0.01 (2/8)	absent (0/12)	absent (0/12)
	ASV145			Parvibaculales	Parvibaculaceae	*Candidate Phaeomarinobacter*	1.11±1.48 (6/12)	absent (0/12)	absent (0/8)	1.32±4.56 (1/12)	2.08±4.09 (5/12)
	ASV726			Sphingomonadales	Sphingomonaceae	*Sphingorhabdus*	0.33±0.53 (5/12)	absent (0/12)	absent (0/8)	0.45±1.44 (2/12)	absent (0/12)

across 3 salinities within each genotype)<br><br>* ASV also detected in the other genotype but only in 1 or 2 salinities	ASV373		Gammaproteobacteria	Cellvibrionales	Halieaceae	nd	0.92±2.36 (8/12)	* 0.16±0.45 (3/12)	absent (0/8)	0.15±0.28 (4/12)	0.06±0.13 (3/12)
	ASV965	Bacteroidota	Bacteroidia	Cytophagales	Cyclobacteriaceae	*Ekhidna*	0.07±0.11 (5/12)	* 0.05±0.18 (2/12)	absent (0/8)	0.36±0.38 (7/12)	absent (0/12)
	ASV59					*Tunicatimonas*	2.86±5.23 (3/12)	absent (0/12)	absent (0/8)	8.18±17.01 (4/12)	absent (0/12)
	ASV409					*Fabibacter*	0.10±0.17 (4/12)	absent (0/12)	absent (0/8)	1.65±2.24 (6/12)	absent (0/12)
	ASV594					*Fabibacter*	absent (0/12)	0.66±0.87 (6/12)	absent (0/8)	absent (0/12)	0.33±0.73 (5/12)
	ASV730					nd	0.07±0.16 (3/12)	absent (0/12)	absent (0/8)	0.84±1.16 (6/12)	absent (0/12)
	ASV171					nd	absent (0/12)	3.13±2.97 (12/12)	absent (0/8)	absent (0/12)	1.05±1.04 (12/12)
	ASV1090					nd	0.08±0.13 (5/12)	absent (0/12)	absent (0/8)	0.03±0.07 (2/12)	absent (0/12)
	ASV704			Flavobacteriales	Flavobacteriaceae	*Muricauda*	absent (0/12)	0.65±0.70 (7/12)	<0.01 (1/8)	absent (0/12)	0.20±0.27 (6/12)
	ASV2797			Chitinophagales	nd	nd	0.04±0.12 (3/12)	absent (0/12)	absent (0/8)	absent (0/12)	absent (0/12)
	ASV106		Rhodothermia	Balneolales	Balneolaceae	*B2706-C7*	0.48±0.57 (7/12)	absent (0/12)	absent (0/8)	7.02±8.67 (6/12)	absent (0/12)

	ASV322	Planctomycetota	Phycisphaerae	Phycisphaerales	Phycisphaeraceae	*SM1A02*	absent (0/12)	2.35±3.90 (4/12)	absent (0/8)	absent (0/12)	0.26±0.90 (1/12)
	ASV1486		Planctomycetes	Pirellulales	Pirellulaceae	*Pir4_lineage*	0.04±0.09 (3/12)	absent (0/12)	absent (0/8)	absent (0/12)	absent (0/12)
	**ASV73**	Actinobacteriota	**Acidimicrobiia**	**Microtrichales**	**Ilumatobacteraceae**	***Ilumatobacter***	***4.47±6.85 (10/12)***	**** 0.04±0.14 (1/12)***	***0.05±0.01 (3/8)***	**0.40±0.62 (5/12)**	**absent (0/12)**
	**ASV277**					***Ilumatobacter***	***0.78±1.02 (5/12)***	***absent (0/12)***	**absent (0/8)**	**absent (0/12)**	**absent (0/12)**
	ASV200		Thermoleophilia	Gaiellales	nd	nd	absent (0/12)	3.07±2.76 (11/12)	<0.01 (n=8) (1/8)	absent (0/12)	0.11±0.19 (4/12)

Supplementary Material References

5. Sauvage, T.; Schmidt, W.E.; Suda, S.; Fredericq, S. A metabarcoding framework for facilitated survey of coral reef and rhodolith endolithic communities with *tuf*A. *BMC Ecol* **2016**, 16, 1-21, DOI: 10.1186/s12898-016-0068-x.

7. Massé, A.; Tribollet, A.; Meziane, T.; Bourguet-Kondracki, M.L.; Yéprémian, C.; Sève, C.; Thiney, N.; Longeon, A.; Couté, A.; Domart-Coulon, I. Functional diversity of microboring *Ostreobium* algae isolated from corals. *Environ Microbiol* **2020**, 22, 4825-4846, DOI: 10.1111/1462-2920.15256.

14. Massé, A.; Domart-Coulon, I.; Golubic, S.; Duché, D.; Tribollet, A. Early skeletal colonization of the coral holobiont by the microboring Ulvophyceae *Ostreobium* sp.. *Sci Rep* **2018**, 8, 1-11, DOI: 10.1038/s41598-018-20196-5.

23. Cárdenas, A.; Raina, J.B.; Pogoreutz, C.; Rädecker, N.; Bougoure, J., Guagliardo, P.; Pernice, M.; Voolstra, C.R. Greater functional diversity and redundancy of coral endolithic microbiomes align with lower coral bleaching susceptibility. *ISME J* **2022**, 16, 1-15. DOI: 10.1038/s41396-022-01283-y.

25. Marcelino, V.R.; van Oppen, M.J.; Verbruggen, H. Highly structured prokaryote communities exist within the skeleton of coral colonies. *ISME J* **2017**, 12, 300-303, DOI: 10.1038/ismej.2017.164.

26. Marcelino, V.R.; Morrow, K.M.; van Oppen, M.J.H.; Bourne, D. G.; Verbruggen, H. Diversity and stability of coral endolithic microbial communities at a naturally high pCO_2 reef. *Mol Ecol* **2017**, 26, 5344-5357, DOI: 10.1111/mec.14268.

48. Lawson, C.A.; Raina, J.B.; Kahlke, T.; Seymour, J.R.; Suggett, D.J. Defining the core microbiome of the symbiotic dinoflagellate, *Symbiodinium*. *Environ Microbiol Rep* **2018**, 10, 7-11, DOI: 10.1111/1758-2229.12599.

49. Camp, E.F.; Kahlke, T.; Nitschke, M.R.; Varkey, D.; Fisher, N.L.; Fujise, L.; Goyen, S.; Hughes, D.J.; Lawson, C.A.; Ros, M.; *et al.* Revealing changes in the microbiome of Symbiodiniaceae under thermal stress. *Environ Microbiol* **2020**, 22, 1294-1309, DOI: 10.1111/1462-2920.14935.

54. Ricci, F.; Tandon, K.; Black, J.R.; Lê Cao, K.A.; Blackall, L.L.; Verbruggen, H. Host Traits and Phylogeny Contribute to Shaping Coral-Bacterial Symbioses. *mSystems* **2022**, 7, e00044-22, DOI: 10.1128/msystems.00044-22.

73. Buerger, P.; Vanstone, R.T.; Maire, J.; van Oppen, M. J. Long-Term Heat Selection of the Coral Endosymbiont *Cladocopium C1acro* (Symbiodiniaceae) Stabilizes Associated Bacterial Communities. *Int J Mol Sci* **2022**, 23, 4913, DOI: 10.3390/ijms23094913.

80. Marcelino, V.R.; Verbruggen, H. Multi-marker metabarcoding of coral skeletons reveals a rich microbiome and diverse evolutionary origins of endolithic algae. *Sci Rep* **2016**, 6, 1-9, DOI: 10.1038/srep31508.

81. Gutner-Hoch, E.; Fine, M. Genotypic diversity and distribution of *Ostreobium* quekettii within scleractinian corals. *Coral Reefs* **2011**, 30, 643-650, DOI: 10.1007/s00338-011-0750-6.

82. Verbruggen, H.; Ashworth, M.; LoDuca, S.T.; Vlaeminck, C.; Cocquyt, E.; Sauvage, T.; Zechman, F.W.; Littler, D.; Littler, M.M. ; Leliaert, F.; *et al.* A multi-locus time-calibrated phylogeny of the siphonous green algae. *Mol Phylogenet Evol* **2009**, 50, 642-653, DOI: 10.1016/j.ympev.2008.12.018.

83. Famà, P.; Wysor, B.; Kooistra, W.H.; Zuccarello, G.C. Molecular phylogeny of the genus *Caulerpa* (Caulerpales, Chlorophyta) inferred from chloroplast *tuf*A gene. *J Phycol* **2002**, 38, 1040–1050, DOI: 10.1046/j.1529-8817.2002.t01-1-01237.x.

84. Callahan, B.J.; McMurdie, P.J.; Rosen, M.J.; Han, A.W.; Johnson, A.J.A.; Holmes, S.P. DADA2: High-resolution sample inference from Illumina amplicon data. *Nature methods* **2016**, 13, 581-583, DOI: 10.1038/nmeth.3869.

85. Yang, S.H.; Tandon, K.; Lu, C.Y.; Wada, N.; Shih, C.J.; Hsiao S.S.Y.; Jane, W.N.; Lee, T.C.; Yang, C.M.; Liu, C.T.; *et al.* Metagenomic, phylogenetic, and functional characterization of predominant endolithic green sulfur bacteria in the coral *Isopora palifera*. *Microbiome* **2019**, 7, 1-13, DOI: 10.1186/s40168-018-0616-z.

86. Neave, M.J.; Rachmawati, R.; Xun, L.; Michell, C.T.; Bourne, D.G.; Apprill, A.; Voolstra, C.R. Differential specificity between closely related corals and abundant *Endozoicomonas* endosymbionts across global scales. *ISME J* **2017**, 11, 186-200, DOI: 10.1038/ismej.2016.95.

87. Pogoreutz, C.; Rädecker, N.; Càrdenas, A.; Gärdes, A.; Wild, C.; Voolstra, C.R. Dominance of *Endozoicomonas* bacteria throughout coral bleaching and mortality suggests structural inflexibility of the *Pocillopora verrucosa* microbiome. *Ecol Evol* **2018**, 8, 2240-2252, DOI: 10.1002/ece3.3830.
